# Effects of glutamine and omega-3 fatty acids on intestinal neomucosa formation on colon serosa in rats

**DOI:** 10.55730/1300-0144.5766

**Published:** 2024-01-05

**Authors:** Mehmet KÖSTEK, Uygar DEMİR, Ramazan UÇAK, Burak Yasin AVCI, Aydın ÜNAL, Osman Bilgin GÜLÇİÇEK, Ozan ÇALIŞKAN, Bülent ÇİTGEZ, Erdinç SERİN, Sıtkı Gürkan YETKİN, Mehmet MİHMANLI, Mehmet ULUDAĞ

**Affiliations:** 1 Department of General Surgery, University of Health Sciences, Şişli Hamidiye Etfal Training and Research Hospital, İstanbul, Turkiye; 2 Department of Pathology, University of Health Sciences, Şişli Hamidiye Etfal Training and Research Hospital, İstanbul, Turkiye; 3 Department of Biochemistry, Amasya University, Sabuncuoğlu Şerefeddin Training and Research Hospital, Amasya, Turkiye; 4 Department of Pediatric Surgery, University of Health Sciences, Bağcılar Training and Research Hospital, İstanbul, Turkiye; 5 Department of General Surgery, University of Health Sciences, Bağcılar Training and Research Hospital, İstanbul, Turkiye; 6 Department of General Surgery, Üsküdar University, Memorial Hospital, İstanbul, Turkiye; 7 Department of Biochemistry, University of Health Sciences, Prof. Dr. Cemil Taşcıoğlu City Hospital, İstanbul, Turkiye

**Keywords:** Glutamine, intestinal neomucosa, short bowel syndrome, omega-3 fatty acids

## Abstract

**Background/aim:**

Intestinal neomucosa formation is a technique defined for the treatment of short bowel syndrome. This study evaluates the effect of glutamine and omega-3 fatty acids on the growth of intestinal neomucosa on the colonic serosal surface has been evaluated.

**Materials and methods:**

Thirty-two adult male Sprague-Dawley rats were randomly divided into 4 groups: sham, control, glutamine, and omega-3. Laparotomy was performed on all groups. For rats other than the sham group, a 1-cm full-thickness incision was made 4 cm proximal to the ileocecal valve, and colonic serosal surface was sutured as a serosal patch over these openings. By using the oral gavage technique, the glutamine group was ingested with 200 mg/kg/day of glutamine, and the omega-3 group was ingested with 100 mg/kg/day of omega-3 fatty acids. At the end of 14 days, the rats were euthanized, blood specimens were collected, and intestinal segments, including serosal patches, were excised.

**Results:**

Transforming growth factor-beta was significantly lower in the glutamine group compared to the control group. Similarly, fibroblast growth factor-2 was significantly lower in the glutamine group compared to the sham group. Intestinal neomucosa formation was observed in 100% of rats in the glutamine group. In the control and omega-3 groups, intestinal neomucosa formation was observed in 57.1% and 60% of rats, respectively. The inflammatory response, granulation tissue formation, and fibroblastic activity were more severe in the rats of the glutamine and omega-3 groups.

**Conclusion:**

The intestinal neomucosa formation is an experimental technique, and both glutamine and omega-3 fatty acids have the potential to positively affect inflammatory response, granulation tissue formation, and fibroblastic activity. Specifically, glutamine has a favorable effect on intestinal neomucosa formation.

## 1. Introduction

Short bowel syndrome is characterized by enterocyte loss due to extensive bowel resection for various reasons. From an anatomical perspective, the length of the remaining small intestine, after the duodenum, is less than 150–200 cm. On the other hand, intestinal insufficiency refers to a condition in which patients are unable to absorb sufficient nutrients and minerals due to short bowel syndrome. Consequently, these patients cannot continue vital functions without additional supportive treatment, and in the case of children, achieving normal development and growth cannot be possible [1–3]. The incidence of short bowel syndrome is approximately 5–10 patients per million per year [1,4]. The most frequent reasons of short bowel syndrome in adults include arterial or venous mesenteric ischemia, chronic enteropathies, surgical complications, Crohn’s disease, volvulus, and trauma. In children, the leading reasons are necrotizing enterocolitis and volvulus due to intestinal malrotation [1–3,5].

Surgical treatments are in a broad spectrum. The aim of surgical treatments is to slow down the intestinal transit time or to expand the intestinal mucosal surface required for absorption. For this purpose, many techniques have been investigated throughout history [6,7]. Another technique that could potentially be used in the treatment of short bowel syndrome is the method of intestinal neomucosa formation. This method aims to close the full thickness defects created in the small intestines by using various body surfaces. It also aims to develop a new mucosal cover on this surface by using the regenerative capacity of the small intestines [2,8].

Glutamine is a precursor for nucleotide synthesis and an important substrate for hepatic gluconeogenesis. Additionally, it is also an important energy source for rapidly dividing cells, such as gastrointestinal tract epithelium, lymphocytes, reticulocytes, and fibroblasts [9,10]. Numerous animal studies have shown that glutamine inhibits mucosal atrophy in the gastrointestinal tract and increases protein synthesis in the gastrointestinal tract mucosa in animals treated with glutamine during gram-negative sepsis [11–14]. It is also effective in maintaining gastrointestinal mucosal glutathione concentrations during ischemia and reperfusion [15]. In short bowel syndrome models, it has been stated that it may contribute to intestinal adaptation [16].

Omega-3 fatty acids are polyunsaturated fatty acids that play an important role in animal lipid metabolism. They are found in vegetable oils as alpha-linolenic acid and in animal oils as docosahexaenoic acid (DHA) and eicosapentaenoic acid (EPA). In many studies, the effects of DHA and EPA, which are abundant in fish oil, on inflammatory diseases and wound healing have been studied. It has been stated that omega-3 fatty acids, known for positive effects in the treatment of atopic dermatitis, rheumatoid arthritis, cyclosporine-related nephrotoxicity, and diabetic nephropathy, also exhibit positive effects in inflammatory bowel diseases [17]. In addition, it was observed in animal experiments that it had a positive effect on wound healing and barrier functions in the intestinal mucosa [18]. In an animal model of induced colitis, omega-3 fatty acids have been shown to stop the inflammation that causes colitis, and in another study, it has been shown to positively affect the healing of colocolic anastomosis [19–21].

Our aim is to examine the effect of glutamine and omega-3 fatty acids on the development of intestinal neomucosa on the colonic serosa.

## 2. Materials and methods

In this study, 32 adult male Sprague-Dawley rats, approximately 10–12 weeks old, with an average weight of 300 ± 30 g, were used. All animals were maintained at room temperature of 22 °C with a 12-h dark/light cycle. All animals in the groups were fed ad libitum with rat chow containing 21% protein and were provided fresh drinking water every day. The study was completed after 14 days. ARRIVE guidelines and EU Directive 2010/63/EU for animal experiments were followed during the experiments [22]. Biochemical and histopathological examinations were performed blindly.

Rats were randomly divided into four groups, with eight rats studied in each group. The procedures were performed under general anesthesia, with 50 mg/kg ketamine (Ketax, Vem Pharmaceuticals, Ankara, Türkiye) and 10 mg/kg xylazine (Control 10%, Doğa Pharmaceutical Company, İstanbul, Türkiye) administered intraperitoneally.

**Figure 1:**
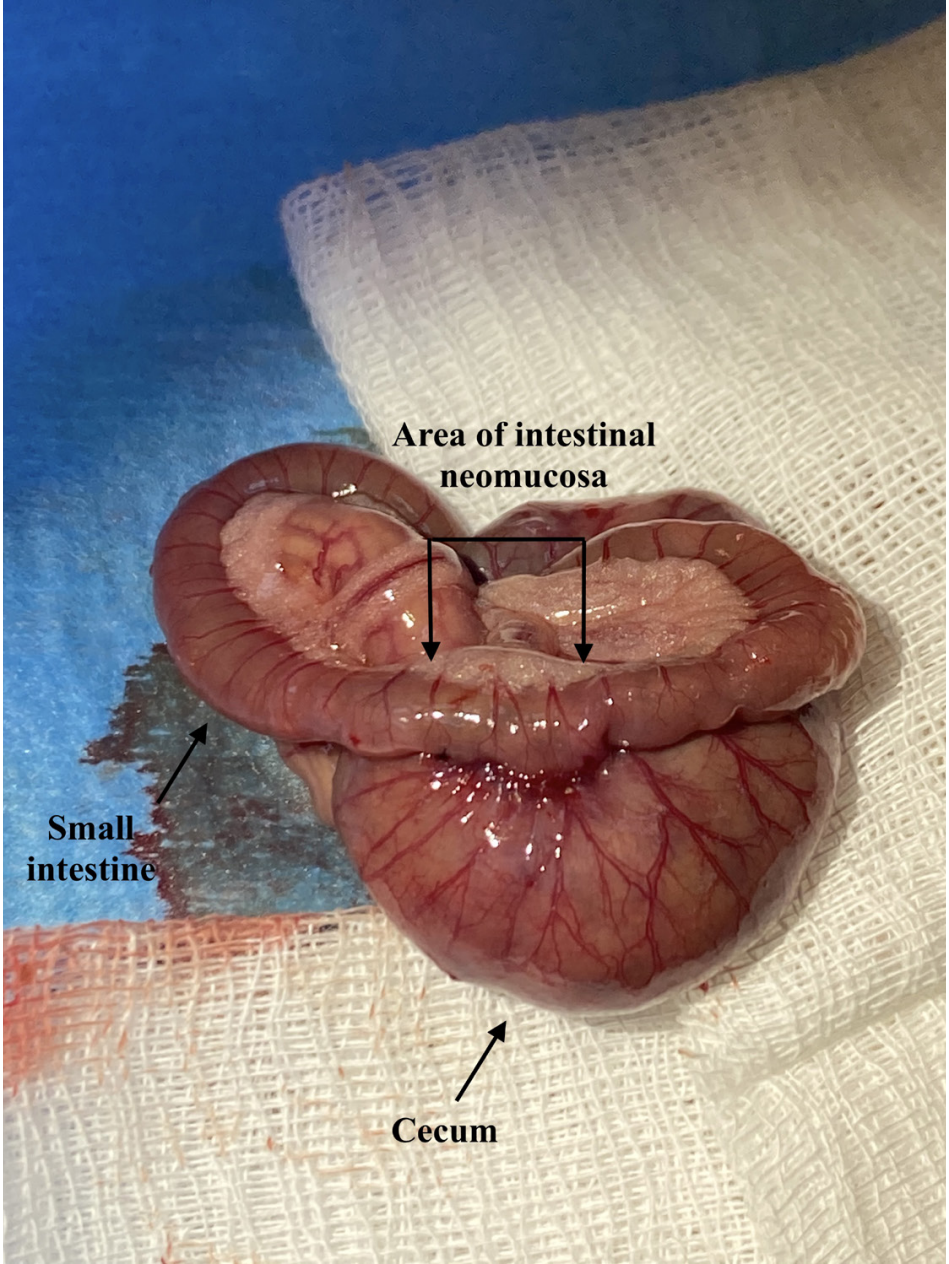
The image after the suturing of the small intestine to the cecum for the growth of intestinal neomucosa.

If necessary, additional doses of these drugs were administered. The rats’ abdomens were shaved, and after providing asepsis and antisepsis with 10% povidone-iodine solution, a 3-cm incision was made in the median line under sterile conditions. In the sham group, the colon and ileum were exposed by manipulation, and the incision was closed without anastomosis. In all other groups (control, glutamine, and omega-3 groups), a 1-cm-long longitudinal full-thickness incision was made on the antimesenteric surface of the small intestine, 4 cm proximal to the ileocecal valve. The ileal defect created was sutured using a continuous technique with 6/0 polypropylene sutures (Doğsan, Türkiye), and the anterior surface of the cecum was covered with a serosal patch (Figure 1). Afterwards, the abdominal organs were positioned, and the anterior abdominal wall was closed continuously with 3/0 silk sutures (Doğsan, Türkiye). The skin was closed, one by one, with 3/0 silk sutures. At the end of the procedure, the incision was once again wiped with povidone iodine, and the operation was completed. A similar technique had been previously employed in the gastric serosa in various studies [23–25].

In the experiment, the glutamine group received 200 mg/kg/day glutamine (Resource Glutamine, Nestle Healthcare Science, Lausanne, Switzerland), the omega-3 group received 100 mg/kg/day of omega-3 fatty acids (EFA-1200, New Life, İstanbul, Türkiye), while the sham and control groups were administered an equivalent volume of physiological saline through gastric gavage for a duration of 14 days, using a rat gavage needle size 16. Glutamine and omega 3 fatty acids were suspended in the solution used for feeding the control rats.

To conclude the experiment at the end of the postoperative 14th day, the rats in the sham group were sacrificed by obtaining their blood via intracardiac puncture under general anesthesia (10 mg/kg xylazine and 50 mg/kg ketamine). On the other hand, relaparotomy was performed under general anesthesia on the 14th postoperative day in animals belonging to the remaining groups. For biochemical analysis, blood was collected via intracardiac puncture and sacrification was performed. Subsequently, the specimen, including the small intestines and the cecum area where the serosal patch was applied, was excised. It was then washed with physiological saline and placed in 10% formaldehyde solution for histopathological examination.

Enzyme-linked immunosorbent assay (ELISA) technique was used to study vascular endothelial growth factor (VEGF), platelet-derived growth factor (PDGF), fibroblast growth factor 2 (FGF2), transforming growth factor-beta (TGF-beta) and epidermal growth factor (EGF) in rat serum. For this technique, the blood collected from the rats was left to clot for a while, then centrifuged at 4000 rpm for 10 min, and the serums were stored at −80 °C after being transferred into Eppendorf tubes. Later, purchased ELISA kits (Sunredbio, Shanghai, China) and sera were run with the BIO-TEK ELx50 (Vermont, USA) washer and BIO-TEK ELx500 (Vermont, USA) reader, and the results were statistically evaluated.

**Figure 2:**
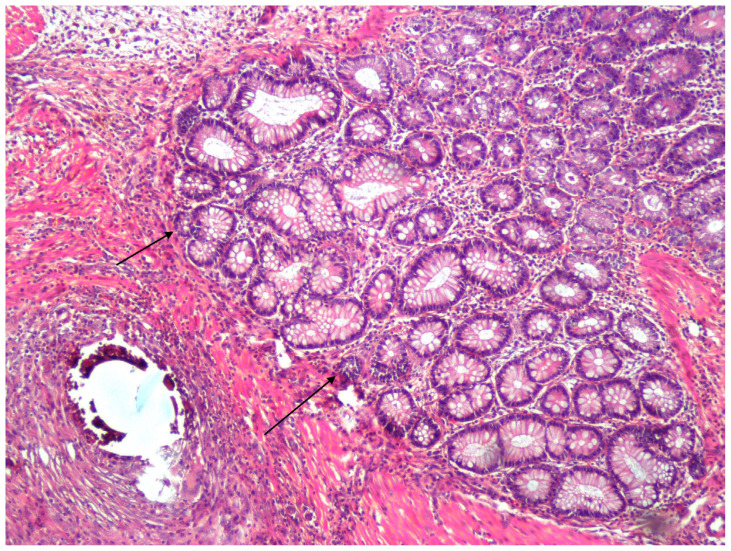
In the glutamine group, new mucosal formation in the center of inflammatory granulation tissue between both mucosae, under the muscle (shown by black arrows) (H.E. ×100).

The materials excised for histopathological examination and kept in 10% formaldehyde solution were cut and embedded in paraffin blocks to examine the neomucosa that would develop on the serosal surface. After the follow-up procedures, the sections with a thickness of 5 μm were stained with hematoxylin-eosin. In these preparations, the inflammatory response, granulation tissue formation, neomucosa formation, angiogenesis, collagen deposition, and fibroblastic activity were studied and scored under light microscopy (Nikon Eclipse E200, Tokyo, Japan) (0: absent, 1: mild, 2: moderate, 3: severe). Mitosis, villus density, and goblet cell count were studied and scored under light microscopy (0: absent, 1: mild, 2: moderate, 3: severe) in the preparations in which neomucosa developed. Cellular density was scored according to the number per 2 mm^2^ (10 high power field (HPF)). (0: absent, 1: 1 cell/2mm^2^ (10 HPF)2: 2–3 cells/2mm^2^ (10 HPF), 3: 4 or above cells/2mm^2^ (10 HPF)). In addition, villi lengths and crypt depths were measured in microns using a light microscope. The obtained data were evaluated statistically (Figure 2).

Statistical analysis was performed using IBM SPSS version 27 (IBM Corporation, Armonk, NY, USA). Results were given as mean ± standard deviation (SD). After determining whether the parameters fit the normal distribution with the Shapiro-Wilk test, the one-way ANOVA test was applied for the normally distributed parametric tests. Tukey posthoc test was used to identify the specific groups that exhibit statistically significant differences in parameters determined by the test results. Kruskal-Wallis test was used for assessing nonparametric distribution. Values with p < 0.05 were considered statistically significant, and posthoc test was applied for paired group comparisons for the parameters that were significant, etc.

## 3. Results

During the study, two rats in the sham group, one in the control group, two in the glutamine group, and three in the Omega-3 group died. In the sham group, one rat died during the operation, while the other rat died on the first postoperative day. This situation was evaluated as a complication after anesthesia. One rat in the control group, one rat in the glutamine group, and two rats in the omega-3 group died due to cannibalism. One rat in the glutamine group and one rat in the omega-3 group died on the postoperative 3rd and 4th days, respectively. No leakage or postoperative mechanical obstruction was detected during the autopsy.

**Figure 3:**
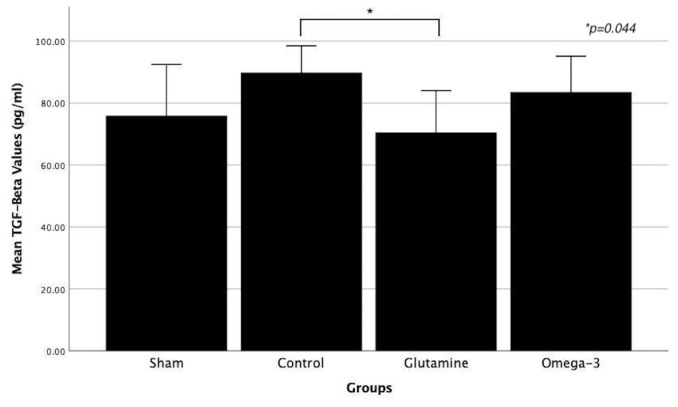
Comparison of serum mean TGF-beta values between groups. Asterisk means statistically significant difference between groups. (TGF-beta: transforming growth factor-beta.)

**Figure 4:**
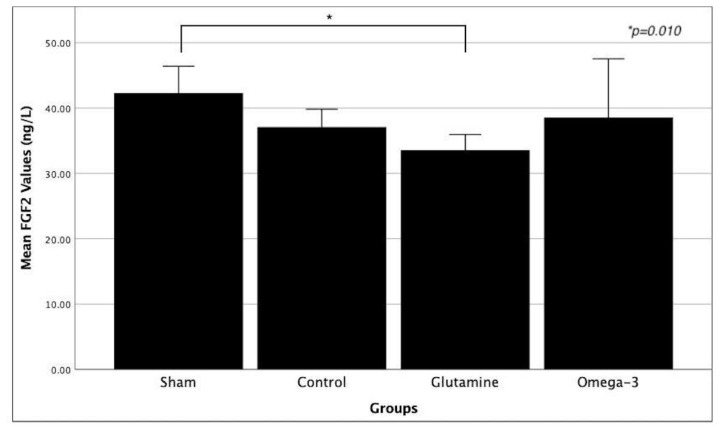
Comparison of serum mean FGF2 values between groups. Asterisk means statistically significant difference between groups. (FGF2: fibroblast growth factor 2.)

Rat serum samples were evaluated with the ELISA test, and test results showed that TGF-beta and FGF2 values were significantly different between the groups. These values are indicated in separate Tables for each group (Table 1). In posthoc analysis, TGF-beta levels were significantly higher in the control group compared to the glutamine group. Additionally, FGF2 levels were significantly higher in the sham group compared to the glutamine group (Figures 3 and 4). No significant differences were observed among the other groups.

The serosal patched areas of rats in the control, glutamine, and omega-3 groups were examined histopathologically and scored for inflammatory response, granulation tissue formation, fibroblastic activity, neomucosa formation, angiogenesis, and collagen deposition (Table 2). The villus density, goblet cell count, and mitosis were scored in rats developing neomucosa, while villus length and crypt depth were measured (Table 3).

The inflammatory response and fibroblastic activity were more severe in the glutamine and omega-3 groups according to the histopathological examination; however, no statistically significant results were obtained. The granulation tissue formation in the sections is examined, and it is observed to be more severe in the glutamine and omega-3 groups compared to the control group. Although there was a significant difference between the groups in the statistical evaluation, no significant difference was found in the posthoc analysis. The neomucosa formation was also examined, revealing that neomucosa formed in all rats in the glutamine group, while it did not form in three rats in the control group and in two rats in the omega-3 group. However, no significant results were obtained in the statistical comparison when the severity of angiogenesis and collagen accumulation was examined; similar responses occurred in all groups.

There was no statistically significant difference, and similar responses were observed in all groups when examining the villus density, mitosis severity, and number of goblet cells on the neomucosa. Additionally, when evaluating the villi lengths and crypt depths on the neomucosa, similar results were seen in all groups, and there was no statistically significant difference.

## 4. Discussion

Intestinal insufficiency and short bowel syndrome are serious medical problems that hinder growth and development due to inadequate absorption and functionality of the intestines, necessitating total parenteral nutrition [26]. The limited success of surgical techniques has led surgeons to seek different techniques [5,6,26,27]. Although intestinal neomucosa formation is an experimental technique, it is a technique that allows for an increase in the intestinal absorption area. With the proliferation of enterocytes, the absorption area increases due to the progression of the small intestinal mucosal surface to the surface used as a patch. The success of this technique depends on the localization of the small intestine to which it is applied, the surface on which the patch is made, the size of the intestinal defect, and the content of nutrients in the lumen. Intestinal neomucosa application can be performed on gastric serosa, colon serosa, peritoneum, or prosthetic materials [2]. However, the application of this experimental technique has not yet been integrated into the clinical stage.

Glutamine, being an essential amino acid for rapidly dividing enterocytes and leukocytes, serves as both an important energy source and a precursor molecule for metabolites. Furthermore, studies have indicated that body glutamine stores are depleted after major operations, necessitating external supplementation is required. Additionally, its effect on intestinal regeneration in rats has been shown in various studies in the literature [28]. Omega-3 fatty acids are essential fatty acids with systemic immunomodulatory effects. They can be characterized as an antiinflammatory food, particularly due to their inhibitory effects on the synthesis of arachidonic acid metabolites [29]. However, studies on the effects of omega-3 fatty acids on the intestinal mucosa are limited.

In this study, we chose to use the 4 cm proximal part of the ileocecal valve and the serosa of the cecum as a patch for intestinal neomucosa formation. Various authors have studied this technique; however, gastric serosa was mostly used in those studies. The cecum serosa was chosen in this study, considering the good blood supply of the cecum serosa and the wider serosal surface in rats as positive aspects for the development of intestinal neomucosa. The 4 cm proximal part of the ileocecal valve was chosen based on its distance from the ileocecal valve, thereby preventing adequate peristalsis in the small intestines. An approximately 1 cm defect was created in the small intestine, resulting in intestinal neomucosa formation on the cecum serosa.

In the biochemical examination, it was revealed that similar results were obtained for VEGF, EGF, and PDGF in all groups, and there was no statistically significant difference. However, there was a significant difference between the control and glutamine groups for TGF-beta. In addition, there is a significant difference between the sham group and the glutamine group for FGF2. TGF-beta is a profibrogenic molecule that regulates the immune system. Although it is also affected by other cytokines in the area of its effects, it generally regulates the maturation and differentiation of immune system cells [30,31]. In this study, it was observed that TGF-beta was significantly lower in the glutamine group compared to the control group. In the literature, it has been shown that glutamine has an antioxidant effect with glutathione and causes a decrease in TGF-beta levels [32]. In another study, the effect of an amino acid mixture containing glutamine, beta-hydroxy beta-methyl butyrate, and arginine on fibrosis caused by radiotherapy was observed. It was found that the TGF-beta value was low in the groups treated with this amino acid mixture [33]. This result obtained in our study may have resulted from the antioxidant effect of glutamine, along with other findings in the literature. FGF2 is a protein that regulates mesenchymal, epithelial, and neuroectodermal cell proliferation. Additionally, it acts as an autocrine growth factor, stimulating intestinal epithelial cell proliferation [34]. In this study, during the comparison between the experimental groups, it was observed that the FGF2 value in the glutamine group was significantly lower than that in the sham group. In the literature, it has been reported that intestinal epithelial repair is stimulated by FGF2 in a TGF-beta-dependent manner in in vitro studies [35,36]. At the same time, considering that the TGF-beta value was significantly lower in the glutamine group compared to the control group in our study, it can be interpreted that the FGF2 value may be lower in proportion to TGF-beta.

In the histopathological examination, there was a notable increase in inflammatory response, granulation tissue formation, and fibroblastic activity observed in both the omega-3 group and in the glutamine group compared to the control group. Although these results are not statistically significant, glutamine and omega-3 fatty acids have favorable effects on inflammatory response, granulation tissue formation, and fibroblastic activity. Additionally, mild or severe neomucosa formation was observed in all rats (100%) in the glutamine group, while no neomucosa formation was observed in three out of seven rats (42.8%) in the control group. Therefore, glutamine has a positive effect on neomucosa formation. Villus density, the amount of mitosis and the number of goblet cells were evaluated qualitatively, while villus length and crypt depth were quantitatively evaluated in rats with neomucosa. However, similar results were obtained between the groups. This indicates that although glutamine contributes to the formation of neomucosa, there is no significant difference between the groups in terms of the properties of the formed neomucosa.

Intestinal neomucosa formation has been studied in various studies. In a previous study, intestinal neomucosa was grown over the gastric serosa in rats, and the effects of glutamine, nesfatin-1, and curcumin on neomucosa were evaluated [24]. In contrast to our findings, increased levels of PDGF, TGF-beta, and VEGF were observed in the glutamine group compared to the control group. Nevertheless, this may be related to the use of colonic serosa or a limited number of animals in this experimental study. There was no statistical difference in the inflammatory process, granulation tissue production, fibroblastic activity, and neomucosa formation between the control group and the glutamine group. However, inflammatory processes, granulation tissue production, and fibroblastic activity were more severe in the glutamine group in our study. In both studies, the authors showed that a high percentage of neomucosa formation was observed in the glutamine group compared to the control group. In a recent study, the effect of glutamine on neomucosa formation was positive; however, it was not statistically significant [37]. The authors showed that oxidative damage was lower and antioxidant enzyme activities were higher in the glutamine group.

There has been no previous study that evaluates the effects of omega-3 fatty acids on neomucosa formation; however, several studies have evaluated the relationship between omega-3 fatty acids and intestinal wound healing. In a study on the healing of colon anastomosis, it was stated that preoperatively started omega-3 fatty acids increased the accumulation of type I collagen on the postoperative 5th day but did not contribute to the tensile strength [20]. In another study conducted on colonic anastomosis, the effect of omega-3 fatty acids, together with ascorbic acid, was examined, and a higher anastomotic opening pressure was observed in the omega-3 group compared to the control group [19]. However, there are studies indicating that intestinal wound healing is delayed due to the inhibitory effect of omega-3 fatty acids on epithelial growth factor receptor transactivation [38]. In our study, inflammatory response, granulation tissue formation and fibroblastic activity was more severe in the omega-3 group. However, there is a need for more studies on the contribution and mechanism of omega-3 fatty acids to intestinal healing and intestinal neomucosa.

The limitations of our study include the loss of animals during the experiment and the limited number of animals that can be used within the framework of ethical rules. Since the treatment responses in rats may be low numerically, conducting intestinal neomucosa formation studies in larger experimental animals such as pigs or rabbits would be a better solution to overcome this limitation.

Intestinal neomucosa formation is a technique that has been studied for the future treatment of patients with short bowel syndrome. Both glutamine and omega-3 fatty acids potentially positively affect inflammatory response, granulation tissue formation, and fibroblastic activity. Glutamine specifically favors the growth of neomucosa on the cecum histopathologically.

## Figures and Tables

**Table 1:** Mean values of growth factors according to groups. Significant values are indicated in bold. (VEGF: vascular endothelial growth factor, PDGF: platelet-derived growth factor, FGF2: fibroblast growth factor 2, TGF-beta: transforming growth factor-beta and EGF: epidermal growth factor.)

	VEGF (ng/mL)	TGF-beta (pg/mL)	EGF (ng/L)	PDGF (ng/mL)	FGF2 (ng/L)
**Sham**	86.45 ± 9.26	75.87 ± 15.77	124.30 ± 50.91	30.88 ± 8.26	42.28 ± 3.91
**Control**	99.75 ± 19.10	89.81 ± 9.37	124.27 ± 33.67	29.07 ± 4.65	37.09 ± 2.94
**Glutamine**	91.82 ± 15.61	70.49 ± 12.91	104.27 ± 32.92	36.38 ± 13.76	33.53 ± 2.30
**Omega-3**	96.60 ± 13.04	83.54 ± 9.31	93.70 ± 28.27	32.99 ± 9.04	38.53 ± 7.24
**p-value**	0.441	**0.049**	0.443	0.554	**0.017**

**Table 2:** Histopathological findings observed in all sections as a result of examinations. Significant values are indicated in bold.

Results of histopathological analysis		Control (n = 7)	Glutamine (n = 6)	Omega-3 (n = 5)	p-value
**Inflammatory response**	None	0	0	0	0.152
	Mild	3 (42.8%)	1 (16.7%)	0	
	Moderate	2 (28.6%)	0	2 (40%)	
	Severe	2 (28.6%)	5 (83.3%)	3 (60%)	
**Granulation tissue formation**	None	1 (14.3%)	0	0	**0.038**
	Mild	3 (42.8%)	1 (16.7%)	0	
	Moderate	2 (28.6%)	0	2 (40%)	
	Severe	1 (14.3%)	5 (83.3%)	3 (60%)	
**Fibroblastic activity**	None	1 (14.3%)	0	0	0.103
	Mild	3 (42.8%)	1 (16.7%)	0	
	Moderate	1 (14.3%)	0	2 (40%)	
	Severe	2 (28.6%)	5 (83.3%)	3 (60%)	
**Neomucosa formation**	None	3 (42.8%)	0	2 (40%)	0.264
	Mild	2 (28.6%)	3 (50%)	1 (20%)	
	Moderate	1 (14.3%)	0	1 (20%)	
	Severe	1 (14.3%)	3 (50%)	1 (20%)	
**Angiogenesis**	None	1 (14.3%)	0	0	0.756
	Mild	1 (14.3%)	0	0	
	Moderate	2 (28.6%)	3 (50%)	3 (60%)	
	Severe	3 (42.8%)	3 (50%)	2 (40%)	
**Collagen deposition**	None	1 (14.3%)	0	0	0.496
	Mild	1 (14.3%)	0	1 (20%)	
	Moderate	3 (42.8%)	3 (50%)	2 (40%)	
	Severe	2 (28.6%)	3 (50%)	2 (40%)	

**Table 3:** Histopathological findings in animals developing neomucosa. Mean villus length and mean crypt depth were expressed as mean ± standard deviation (lowest value-highest value).

Histopathological analysis of neomucosa		Control (n = 4)	Glutamine (n = 6)	Omega-3 (n = 3)	p-value
**Villus density**	Mild	2 (50%)	3 (50%)	1 (33.3%)	1
	Moderate	0	0	1 (33.3%)	
	Severe	2 (50%)	3 (50%)	1 (33.3%)	
**Mitosis**	Mild	0	0	0	0.888
	Moderate	2 (50%)	3 (50%)	2 (66.7%)	
	Severe	2 (50%)	3 (50%)	1 (33.3%)	
**Goblet cell count**	Mild	0	3 (50%)	1 (33.3%)	0.162
	Moderate	2 (50%)	1 (16.7%)	2 (66.7%)	
	Severe	2 (50%)	2 (33.3%)	0	
**Mean villus lenght (micron)**		4.37 ± 2.14 (1.5–6)	2.67 ± 1.51 (1–5)	3.17 ± 1.76 (1.5–5)	0.359
**Mean crypt depth (micron)**		7.62 ± 6.18 (1.5–16)	6.50 ± 3.83 (3–12)	7.17 ± 5.30 (1.5–12)	0.938

## References

[1] Billiauws L Maggiori L Joly F Panis Y Medical and surgical management of short bowel syndrome Journal of Visceral Surgery 2018 155 4 283 291 10.1016/j.jviscsurg.2017.12.012 30041905

[2] Freud E Eshet R Insights from animal models for growing intestinal neomucosa with serosal patching - a still untapped technique for the treatment of short bowel syndrome Laboratory Animals 2001 35 2 180 187 10.1258/0023677011911453 11315169

[3] Amiot A Messing B Corcos O Panis Y Joly F Determinants of home parenteral nutrition dependence and survival of 268 patients with non-malignant short bowel syndrome Clinical Nutrition 2013 32 3 368 374 10.1016/j.clnu.2012.08.007 22992308

[4] Carbonnel F Cosnes J Chevret S Beaugerie L Ngô Y The role of anatomic factors in nutritional autonomy after extensive small bowel resection JPEN Journal of Parenteral and Enteral Nutrition 1996 20 4 275 280 10.1177/0148607196020004275 8865109

[5] Lauro A Lacaille F Short bowel syndrome in children and adults: from rehabilitation to transplantation Expert Review of Gastroenterology and Hepatology 2019 13 1 55 70 10.1080/17474124.2019.1541736 30791840

[6] Sommovilla J Warner BW Surgical options to enhance intestinal function in patients with short bowel syndrome Current Opinion in Pediatrics 2014 26 3 350 355 10.1097/MOP.0000000000000103 24759225 PMC5167627

[7] Millar AJ Non-transplant surgery for short bowel syndrome Pediatric Surgery International 2013 29 10 983 987 10.1007/s00383-013-3390-9 23982389

[8] Bragg LE Thompson JS The influence of serosal patch size on the growth of small intestinal neomucosa Journal of Surgical Research 1986 40 5 426 431 10.1016/0022-4804(86)90210-6 3090369

[9] Shen Y Zhang Y Li W Chen K Xiang M Glutamine metabolism: from proliferating cells to cardiomyocytes Metabolism 2021 121 154778 10.1016/j.metabol.2021.154778 33901502

[10] Chen Y Tsai YH Tseng BJ Tseng SH Influence of Growth Hormone and Glutamine on Intestinal Stem Cells: A Narrative Review Nutrients 2019 11 8 1941 10.3390/nu11081941 31426533 PMC6724402

[11] Tamada H Nezu R Imamura I Matsuo Y Takagi Y The dipeptide alanyl-glutamine prevents intestinal mucosal atrophy in parenterally fed rats JPEN Journal of Parenteral and Enteral Nutrition 1992 16 2 110 116 10.1177/0148607192016002110 1372946

[12] Burrin DG Shulman RJ Langston C Storm MC Supplemental alanylglutamine, organ growth, and nitrogen metabolism in neonatal pigs fed by total parenteral nutrition JPEN Journal of Parenteral and Enteral Nutrition 1994 18 4 313 319 10.1177/014860719401800406 7933437

[13] Yoshida S Leskiw MJ Schluter MD Bush KT Nagele RG Effect of total parenteral nutrition, systemic sepsis, and glutamine on gut mucosa in rats American Journal of Physiology-Endocrinology and Metabolism 1992 263 2 Pt 1 E368 373 10.1152/ajpendo.1992.263.2.E368 1514620

[14] Higashiguchi T Hasselgren PO Wagner K Fischer JE Effect of glutamine on protein synthesis in isolated intestinal epithelial cells JPEN Journal of Parenteral and Enteral Nutrition 1993 17 4 307 314 10.1177/0148607193017004307 8271353

[15] Başoglu M Yildirgan I Akçay F Kiziltunç A Kavak I Glutathione and nitric oxide concentrations in glutamine-infused rabbits with intestinal ischaemia/reperfusion European Journal of Clinical Chemistry and Clinical Biochemistry 1997 35 6 415 419 10.1515/cclm.1997.35.6.415 9228323

[16] Duggan C Gannon J Walker WA Protective nutrients and functional foods for the gastrointestinal tract American Journal of Clinical Nutrition 2002 75 5 789 808 10.1093/ajcn/75.5.789 11976152

[17] Hickman MA Interventional nutrition for gastrointestinal disease Clinical Techniques in Small Animal Practice 1998 13 4 211 216 10.1016/S1096-2867(98)80005-4 9842113

[18] Mukherjee K Kavalukas SL Barbul A Nutritional Aspects of Gastrointestinal Wound Healing Advances in Wound Care (New Rochelle) 2016 5 11 507 515 10.1089/wound.2015.0671 PMC5105338 27867755

[19] Ekçi B Karabicak I Atukeren P Altinlio E Tomaoglu K The effect of omega-3 fatty acid and ascorbic acid on healing of ischemic colon anastomoses Annali Italiani di Chirurgia 2011 82 6 475 479 22229237

[20] de Castilho TJ Campos AC Mello EV Effect of omega-3 fatty acid in the healing process of colonic anastomosis in rats Arquivos Brasileiros di Cirurgia Digestiva (Sao Paulo) 2015 28 4 258 261 10.1590/s0102-6720201500040010 PMC4755178 26734796

[21] Hudert CA Weylandt KH Lu Y Wang J Hong S Transgenic mice rich in endogenous omega-3 fatty acids are protected from colitis Proceedings of the National Academy of Sciences USA 2006 103 30 11276 11281 Epub 2006 Jul 17. 10.1073/pnas.0601280103 PMC1544078 16847262

[22] Kilkenny C Browne WJ Cuthill IC Emerson M Altman DG Improving bioscience research reporting: the ARRIVE guidelines for reporting animal research PLoS Biology 2010 8 6 e1000412 10.1371/journal.pbio.1000412 20613859 PMC2893951

[23] Adas G Adas M Arikan S Sarvan AK Toklu AS Effect of growth hormone, hyperbaric oxygen and combined therapy on the gastric serosa World Journal of Gastroenterology 2013 19 19 2904 2912 10.3748/wjg.v19.i19.2904 23704823 PMC3660815

[24] Gulcicek OB Solmaz A Yiğitbaş H Ercetin C Yavuz E Comparison of the Effects of Glutamine, Curcumin, and Nesfatin-1 on the Gastric Serosal Surface Neomucosa Formation: An Experimental Rodent Model Gastroenterology Research and Practice 2016 2016 2081962 10.1155/2016/2081962 27525002 PMC4972927

[25] Binboğa S Kasapoğlu P Binboğa E Cikot M Baytekin F Effects of platelet rich plasma on the gastric serosal surface neomucosa formation: an experimental rodent model Turkish Journal of Biochemistry 2019 44 1 32 40 10.1515/tjb-2018-0098

[26] Yap JYK Roberts AJ Bines JE Paediatric intestinal failure and transplantation Journal of Paediatrics and Child Health 2020 56 11 1747 1753 10.1111/jpc.15052 33197983

[27] Sudan D Thompson J Botha J Grant W Antonson D Comparison of intestinal lengthening procedures for patients with short bowel syndrome Annals of Surgery 2007 246 4 593 604 10.1097/SLA.0b013e318155aa0c 17893496

[28] Ellinger S Can specific nutrients stimulate bowel wound healing? Current Opinion in Clinical Nutrition and Metabolic Care 2016 19 5 371 376 10.1097/MCO.0000000000000303 27348151

[29] Infante M Fabbri A Della-Morte D Ricordi C The importance of vitamin D and omega-3 PUFA supplementation: a nonpharmacologic immunomodulation strategy to halt autoimmunity European Review for Medical and Pharmacological Sciences 2022 26 18 6787 6795 10.26355/eurrev_202209_29780 36196727

[30] Letterio JJ Roberts AB Regulation of immune responses by TGF-beta Annual Reviews in Immunology 1998 16 137 161 10.1146/annurev.immunol.16.1.137 9597127

[31] Keski-Oja J Leof EB Lyons RM Coffey RJ Jr Moses HL Transforming growth factors and control of neoplastic cell growth Journal of Cellular Biochemistry 1987 33 2 95 107 10.1002/jcb.240330204 3553215

[32] Shrestha N Chand L Han MK Lee SO Kim CY Glutamine inhibits CCl4 induced liver fibrosis in mice and TGF-β1 mediated epithelial-mesenchymal transition in mouse hepatocytes Food and Chemical Toxicology 2016 93 129 137 10.1016/j.fct.2016.04.024 27137983

[33] Yavas C Yavas G Celik E Buyukyoruk A Buyukyoruk C Beta-Hydroxy-Beta-Methyl-Butyrate, L-glutamine, and L-arginine Supplementation Improves Radiation-Induce Acute Intestinal Toxicity Journal of Dietary Supplements 2019 16 5 576 591 10.1080/19390211.2018.1472709 29969326

[34] Dignass AU Sturm A Peptide growth factors in the intestine European Journal of Gastroenterology and Hepatology 2001 13 7 763 770 10.1097/00042737-200107000-00002 11474304

[35] Dignass AU Tsunekawa S Podolsky DK Fibroblast growth factors modulate intestinal epithelial cell growth and migration Gastroenterology 1994 106 5 1254 1262 10.1016/0016-5085(94)90017-5 7513666

[36] Paimela H Goddard PJ Carter K Khakee R McNeil PL Restitution of frog gastric mucosa in vitro: effect of basic fibroblast growth factor Gastroenterology 1993 104 5 1337 1345 10.1016/0016-5085(93)90342-A 8482448

[37] Akbaş A Gülcicek OB Yavuz E Yigitbas H Solmaz A Effect of glutamine use on the formation of intestinal neomucosa on peritoneal surface in rats Turkish Journal of Trauma and Emergency Surgery 2022 28 11 1541 1548 10.14744/tjtes.2022.36903 36282159 PMC10277354

[38] Turk HF Monk JM Fan YY Callaway ES Weeks B Inhibitory effects of omega-3 fatty acids on injury-induced epidermal growth factor receptor transactivation contribute to delayed wound healing American Journal of Physiology-Cell Physiology 2013 304 9 C905 917 10.1152/ajpcell.00379.2012 23426968 PMC3651607

